# Grazing mediates microclimate effects on lichen performance near its warm-range margin

**DOI:** 10.1093/aob/mcag064

**Published:** 2026-03-28

**Authors:** Sonia Merinero, Caroline Greiser, Kristoffer Hylander, Alicia Valdés, Johan Ehrlén

**Affiliations:** Departamento de Biología, Universidad Rey Juan Carlos, Móstoles 28933, Spain; Instituto de Investigación en Cambio Global (IICG-URJC), Universidad Rey Juan Carlos, Móstoles 28933, Spain; Department of Ecology, Environment and Plant Sciences & Bolin Centre for Climate Research, Stockholm University, Stockholm SE-106 91, Sweden; Department of Physical Geography, Stockholm University, Stockholm SE-106 91, Sweden; Department of Forest Ecology and Management, Swedish University of Agricultural Sciences, Umeå SE-901 83, Sweden; Department of Ecology, Environment and Plant Sciences & Bolin Centre for Climate Research, Stockholm University, Stockholm SE-106 91, Sweden; Biodiversity Research Institute (IMIB), University of Oviedo–CSIC–Principality of Asturias, Mieres, Asturias 33600 Spain; Department of Organismal and Systems Biology, University of Oviedo, Oviedo, Asturias 33071 Spain; Department of Ecology, Environment and Plant Sciences & Bolin Centre for Climate Research, Stockholm University, Stockholm SE-106 91, Sweden

**Keywords:** Common garden transplant experiment, forest lichen, intraspecific variation, microclimate, mollusc grazing, *Peltigera aphthosa*, plant–climate interactions, plant–herbivore interactions, population differentiation

## Abstract

**Background and Aims:**

Climate influences species performance and distribution both directly, through physiological constraints, and indirectly, by modulating biotic interactions. However, the relative importance of these pathways remains poorly understood, limiting our ability to predict species responses to environmental change. Here, we investigated how microclimate influences the growth of a cold-adapted lichen both directly and indirectly, through effects on mollusc grazing, and whether these effects differ among populations of different origins.

**Methods:**

We conducted a transplant experiment using lobes of *Peltigera aphthosa* collected from five geographically distant populations along a 1200-km latitudinal gradient in Sweden. The lobes were transplanted to 56 forest sites spanning a broad microclimatic gradient near the species' warm-range margin in Sweden, and we monitored their growth and grazing damage for one year. Using piecewise structural equation models, we quantified the direct and indirect effects of microclimate through grazing on lichen growth and assessed whether these effects differed among source populations.

**Key Results:**

We found no evidence for direct effects of microclimate on lichen growth. Instead, microclimate indirectly influenced growth through mollusc grazing. Grazing damage increased with warmer temperatures (more growing degree days) and higher air humidity (lower vapour pressure deficit) during the growing season and grazing reduced lichen growth. While population origin did not affect direct responses to microclimate, populations differed in their susceptibility to grazing.

**Conclusions:**

Our study highlights the importance of indirect microclimatic effects mediated by biotic interactions in shaping species performance near its warm-range margin. The absence of direct microclimatic effects, combined with intraspecific variation in susceptibility to grazing, underscores the need to consider both biotic interactions and differences among populations when predicting species responses to climate change. Transplant experiments across microclimatic gradients offer a valuable method to gain insights into the complex interplay between local adaptation and abiotic and biotic factors of species performance.

## INTRODUCTION

Climate is a key abiotic factor driving species distributions and range limits through its effects on physiological tolerance, population dynamics, abundance and persistence (Gaston, 2003; Ehrlén and Morris, 2015). Growing evidence shows that indirect effects of climate, mediated through biotic interactions, can also be equally or even more influential in shaping species performance and distribution patterns (Wisz *et al.*, 2013; Ockendon *et al.*, 2014; Morris *et al.*, 2020; Christiansen *et al.*, 2024). Climate change may thus negatively affect a species not only through physiological constraints but also by enhancing the success of antagonists (e.g. herbivores, pathogens) or disrupting mutualistic relationships (e.g. with pollinators or mycorrhizal fungi) (Gellesch *et al.*, 2013). The intensity of biotic interactions often varies systematically across climatic gradients, with biotic pressure being stronger at the warm low-latitude margins of the ranges of cold-adapted species, while climatic pressure dominates at colder, high-latitude margins (Schemske *et al.*, 2009; Paquette and Hargreaves, 2021; but see Louthan *et al.*, 2025). Indeed, antagonistic trophic interactions such as herbivory often intensify with temperature and precipitation (Rodríguez-Castañeda, 2013). Advancing our understanding of the relative importance of the direct and indirect pathways through which climate affects species performance is essential to predict ecological responses to climate change and guiding conservation priorities (Louthan *et al.*, 2025), not least in cold-adapted species, which may be especially sensitive to changing climatic and biotic pressures (Mäkinen *et al.*, 2025).

The direct and indirect effects of climate are unlikely to be uniform across a species’ range, since populations often differ in their responses to both climate and biotic factors due to genetic differentiation and local adaptation (Clausen *et al.*, 1940; Leimu and Fischer, 2008; Fornoni, 2011). Cold-adapted plant populations, for instance, may be more negatively affected by warming than those from warmer areas (e.g. Kreyling *et al.*, 2014; Merinero *et al.*, 2020). Similarly, resistance to herbivory can vary among populations. Populations exposed to intense herbivory typically develop stronger defensive traits than those experiencing weaker pressure (Karban and Myers, 1989; Fornoni, 2011; Züst *et al.*, 2012). Consequently, across a species’ distribution range, concurrent gradients in climate and herbivory can have very different effects. Understanding how such intraspecific variation influences the interplay between climate and biotic interactions remains a critical challenge for predicting species’ responses under ongoing climate change.

Experimental approaches constitute a powerful tool to disentangle the relative contributions of abiotic and biotic factors to species performance. Transplant experiments are particularly valuable, as they allow the assessment of local adaptation by comparing responses among populations across environmental gradients (Clausen *et al.*, 1940; Blanquart *et al.*, 2013). When replicated across multiple field common gardens, these experiments can reveal how populations respond to simultaneous variation in abiotic and biotic factors (de Villemereuil *et al.*, 2016). However, studies explicitly testing the effects of multiple environmental factors across populations remain scarce (but see e.g. Merinero *et al.*, 2020; Ramírez-Valiente *et al.*, 2022). Such experiments should accurately characterize the climatic conditions that organisms experience. In forest ecosystems, microclimatic conditions can differ substantially from regional climate averages due to canopy buffering effects (De Frenne *et al.*, 2019). Locally measured microclimate data are therefore essential for understanding the ecology of forest understorey species, and provide more ecologically relevant insights than downscaled macroclimatic data (Kemppinen *et al.*, 2024).

Forest lichens represent an excellent system to test the interplay of microclimate and biotic interactions on species performance. Their poikilohydric physiology makes them highly sensitive to fine-scale variation in temperature, humidity and light (reviewed by Stanton *et al.*, 2023). For example, some cold-adapted species show strong declines in growth and survival under warm and dry conditions (Bjerke, 2011; Colesie *et al.*, 2018; Esseen *et al.*, 2022). Lichens also experience strong biotic pressures, particularly from mollusc grazing, which varies depending on species-specific chemical and structural traits (Asplund and Wardle, 2013). Mollusc grazing can influence lichen distribution on local and regional scales (Gauslaa, 2008; Asplund *et al.*, 2010). The effects of climate and grazing may not be uniform across a species range, as populations adapted to different environmental conditions may vary in both their climate sensitivity and vulnerability to grazers. Although intraspecific variation in climate sensitivity and palatability has been documented for several species (e.g. Sonesson *et al.*, 2007; Asplund, 2011; Dal Grande *et al.*, 2017), the combined effect of climatic and biotic factors across populations remains largely unexplored in lichens.

Here, we investigate how microclimate influences the growth of the cold-adapted ground-dwelling lichen *Peltigera aphthosa* through both direct effects and indirect effects mediated by mollusc grazing, and whether these effects vary among source populations distributed along a latitudinal gradient. To address this, we transplanted lichens from five geographically distant populations collected along a 1200-km latitudinal gradient in Sweden into 56 forest sites spanning a broad microclimatic gradient near the warm-range margin of the species in Sweden. In this region, the abundance of molluscs is expected to be higher than in the species’ core and cold-range areas (Neubert *et al.*, 2019). We combined this design with *in situ* microclimate measurements to capture the actual temperature and humidity conditions experienced by the transplants. Although the transplant area represents smaller climatic variation than the total climatic range of the source populations, we expect that the lichen responses to this local variation are qualitatively similar to those that would occur across larger spatial scales. By using distant populations across the species range, we integrated potential local adaptation into our experimental design, testing whether genetic differentiation and prior adaptation to local abiotic and biotic factors influence populations’ performance under different microclimatic conditions. We did not exclude molluscs from the experiment, as our objective was to quantify how microclimate shapes lichen performance directly and through biotic interactions under natural conditions, rather than to assess the effects of microclimate as they would have been if molluscs were absent. The use of piecewise structural equation models (pSEMs) allowed us to statistically partition the direct microclimatic effects from the indirect ones mediated by mollusc grazing.

**Fig. 1.**
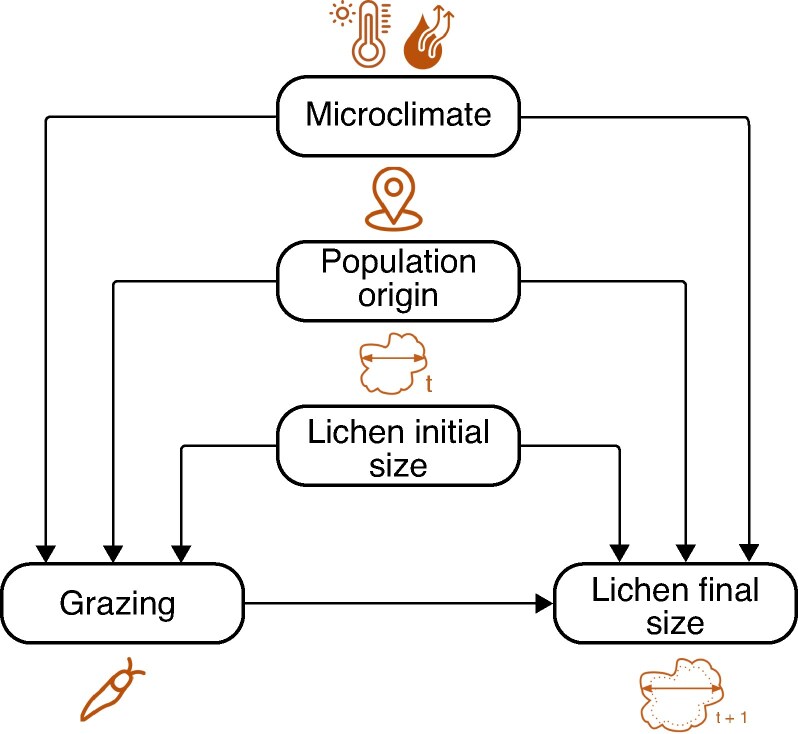
Conceptual model with predicted direct effects of microclimate and its indirect effects through grazing damage on lichen final size (as measured in lichen area, biomass and specific thallus mass). The origin of the population is expected to influence the final size of the lichen directly and indirectly through differential susceptibility to grazing among populations. The initial size of the lichen (area and biomass in time t) is included as a covariate for the final size of the lichen (area and biomass in time t+1) and the grazing damage. Icons are sourced from Flaticon.com and original illustrations are by the authors.

We predicted that microclimate affects lichen growth both directly and indirectly through mollusc grazing, and that these effects differ among source populations (Fig. 1). Specifically, we hypothesize that: (1) lichen growth increases directly with humidity and temperature and declines under dry, cold conditions; (2) indirect negative effects of humid and warm conditions on growth are mediated by increased mollusc grazing; and (3) populations differ in their direct responses to microclimate and their sensitivity to mollusc grazing, with northern populations performing relatively better under colder conditions and being more negatively affected by grazing, reflecting local adaptation to their native colder environments with lower grazing pressure.

## MATERIALS AND METHODS

### Study species

**Fig. 2.**
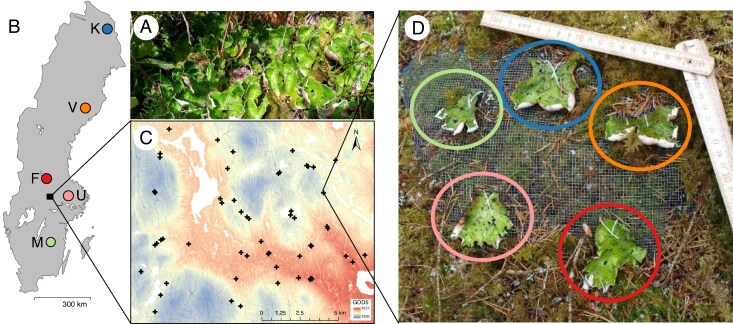
Experimental design. (A) Mat of *Peltigera aphthosa*. (B) Locations of the five source populations, represented with different colours along a 1200-km latitudinal gradient in Sweden (K, Kiruna; V, Vindeln; F, Fagersta; U, Uppsala; M, Malexander). (C) Location of the 56 field common gardens (black crosses) in a 16 × 16 km landscape within the transitional area of the boreal and temperate forest in Sweden. Background map corresponds to growing degree days at 5 °C (GDD5) at a resolution of 50 m extracted from Meineri and Hylander (2017). (D) Example of a transplant unit with five lobes of *P. aphthosa* corresponding to each source population (the scale is in centimetres).

The lichen *Peltigera aphthosa* is considered a cold-adapted species because it is common in artic, boreal and, more rarely, in temperate zones in the Northern Hemisphere (Global Biodiversity Information Facility (GBIF): https://www.gbif.org/species/2601143). This circumpolar species occurs in coniferous, deciduous and mixed forests, as well as in bogs and shrub tundra, typically growing on soil among bryophytes and, less frequently, on bare, acidic mineral soils (Ahti *et al.*, 2007). In Sweden it is frequent in the northernmost and central regions, occurring more scattered towards the southernmost parts of the country (www.artdatabanken.se). This foliose lichen forms compact to widely loose mats composed of multiple 2- to 5-cm wide lobes with apical growth (Fig. 2A). Although the main photobiont of lichen is a green alga (*Coccomyxa* sp.), *P. aphthosa* is a nitrogen-fixing species due to the presence of cyanobacterial colonies (*Nostoc* sp.), visible as black spots on the surface of the thallus (Fig. 2A). This species is a relatively fast-growing lichen, with up to 40 % increases in biomass and area expansion during summer in northern latitudes (Dahlman *et al.*, 2002; Palmqvist *et al.*, 2017), and produces nitrogen- and carbon-based secondary metabolites that may deter grazing (Kaasalainen *et al.*, 2012; Asplund and Wardle, 2015). Because *P. aphthosa* experiences very different environmental conditions throughout its distribution range in Sweden, genetic differentiation among populations is likely to occur.

### Material collection

We collected lobes of *P. aphthosa* from five geographically distant populations across a 1200-km latitudinal gradient in Sweden in August 2016, covering the species distribution range and encompassing substantial variation in climate (Fig. 2B, Supplementary Data Table S1). To maximize potential genetic differences, populations were separated by several hundred kilometres. In each population, we collected 70 sterile lobes of a representative size from one or more mats (depending on the size and number of mats) and removed debris in the laboratory.

### Transplant area and experiment

We transplanted *P. aphthosa* lobes from the five source populations to 56 sites differing in microclimatic conditions using a replicated field common garden design within a landscape of about 16 × 16 km in central–southern Sweden, relatively close to the species warm-range margin (Västmanland and Dalarna counties, 59°56′N, 15°30′E, 280 m a.s.l.) (Fig. 2C). The study area lies within the boreal–temperate forest transition zone, characterized by pronounced spatial heterogeneity in climate, soils and vegetation (Sjörs, 1999). The regional climate is humid and cold–temperate, with cold winters and temperate summers. The mean annual temperature is ∼6 °C and annual precipitation averages around 600 mm, falling predominantly in summer and as snow during winter (SMHI, 2024). The growing season typically extends from April to September. Due to the complex topography and forest structure, local microclimatic conditions vary substantially across the landscape, with differences in canopy cover, elevation and soil moisture contributing to fine-scale variation in temperature and humidity (Greiser *et al.*, 2021). Vegetation is dominated by managed coniferous forests, primarily composed of Scots pine (*Pinus sylvestris*) and Norway spruce (*Picea abies*), interspersed with scattered deciduous trees such as *Betula* spp. The forest floor is typically covered by bryophytes, dwarf shrubs and lichens, with herbaceous plants occurring in moist and nutrient-rich microsites.

To capture the full range of microclimatic variation relevant to lichen performance, we used a stratified site selection approach based on high-resolution (50 m) gridded environmental data, including forest inventory data (provided by the company Sveaskog) and temperature data (Meineri and Hylander, 2017). This allowed us to select sites spanning broad gradients of microclimate, forest type and canopy openness (see further details on site selection criteria in Greiser *et al.*, 2021). Prior data on mollusc abundance in the study area were unavailable, and we assumed that grazing pressure would vary across the landscape as a function of environmental heterogeneity. While all selected sites appeared ecologically suitable for the focal lichen species, *P. aphthosa* was absent from the specific transplant sites, though present in the study region (the nearest record at ∼18 km away; Artportalen: www.artdatabanken.se). We established transplant units in all 56 sites in September 2016. Each unit consisted of a 30 × 30 cm nylon net with five lobes of *P. aphthosa*, one from each source population, resulting in a total of 350 lobes (Fig. 2D). Lobes were randomly assigned to nets and fastened at the base with white polyester thread, allowing natural curling upon desiccation. One unit was randomly placed at 44 sites and two units at 14 sites, resulting in a total of 70 transplant units. We were unable to place two replicate units at all sites as the availability of lobes from some source populations was limited. At each site, we cleared a 50 × 50 cm plot and placed a locally collected cushion of the feather moss *Hylocomium splendens*. The transplant net was placed over the moss and anchored with plastic-coated metal wire. To simulate natural growth conditions, three to five shoots of *H. splendens* were placed below each lobe (Fig. 2D). After 1 year, 334 of the 350 lobes were recovered, with no evidence of population-specific loss among the 16 missing lobes.

### Performance measurements

Lichen growth can be assessed using various metrics that capture distinct physiological processes influenced by environmental conditions. Typically, humidity drives surface area expansion, while temperature and light play a key role in biomass accumulation (Palmqvist, 2000; Larsson *et al.*, 2012). We evaluated transplant performance by measuring changes in lobe area and biomass for 1 year (September 2016–September 2017). All measurements were carried out in the same way before and after transplantation. First, to record lobe area (cm^2^), lobes were hydrated with deionized water, scanned using a digital scanner (Epson Perfection 4870), and their area was estimated by manually delineating the images in ImageJ (v1.51.r). Second, to record biomass (DM, mg), the lobes were air-dried for 48 h and weighed. Ten extra control lobes, treated identically, were oven-dried (60 °C for 72 h) to determine a dry mass correction factor, which was applied to estimate the oven-dry mass of the transplants. Finally, we calculated the specific thallus mass (STM; mg DM cm^−2^), a proxy for lobe thickness analogous to the specific leaf area (SLA) in plant functional ecology. Increased STM correlates with higher resource allocation, water retention and enhanced photosynthetic efficiency, and has been shown to shift substantially in response to changes in light exposure and temperature (Larsson *et al.*, 2012; Merinero *et al.*, 2015; Borge and Ellis, 2024).

### Grazing damage

At the end of the experiment, we visually estimated grazing damage on each transplant using a five-category scale based on the proportion of grazed tissue: (a) 0–1 %, (b) 2–11 %, (c) 11–20 %, (d) 20–50 %, (e) 50–90 % (Supplementary Data Fig. S1; adapted from Benesperi and Tretiach, 2004; Greiser *et al.*, 2021). For analysis, categories were converted to their respective mean value (a = 0.5 %, b = 6.5 %, c = 15.5 %, d = 35 %, e = 70 %) and were expressed as proportions from 0 to 1.

### Environmental data

To characterize microclimate conditions, we installed two temperature loggers (Maximum Integrated iButton, DS1923 or DS1921G-F5) per site, recording every 3 h. One logger was placed 5–10 cm above ground inside an inverted white plastic cup on a wooden stick. A second logger, also recording relative humidity (%), was mounted 1 m above ground inside a horizontal white PVC tube (∼25 cm) attached to a tree trunk (Greiser *et al.*, 2021). Both casings shielded the loggers from rain and direct sunlight. From these data (September 2016–September 2017) we derived two indices. We calculated two climatic indices relevant to the ecophysiological performance (Greiser *et al.*, 2021; Stanton *et al.*, 2023): (1) growing degree days (GDD) for the whole period based on the ground-logger data and using a threshold of 5 °C, and (2) maximum vapour pressure deficit (VPD), i.e. air dryness, during spring and summer (May–August 2017) using temperature and humidity data from the 1-m logger [see Greiser *et al.* (2021) for more details on predictor computation]. To estimate canopy openness (as a proxy for summer light availability), we took hemispherical photos at 50 cm above ground using a fisheye lens (Aukey 360° PL-F2) mounted on a smartphone (BQ Aquaris E5). The images were analysed with Gap Light Analyzer software (v2.0; Frazer *et al.*, 1999).

### Statistical analyses

All statistical analyses were conducted in R v.4.1.4 (R Core Team, 2022). We used structural equation modelling to test our hypotheses regarding the direct and indirect (grazing-mediated) effects of microclimate on lichen performance and how these effects vary among populations. Specifically, we constructed three piecewise structural equation models (pSEMs) using the psem package (Lefcheck, 2016), one for each performance measurement: final lobe area, biomass and STM. Each piecewise SEM included two submodels: a submodel with final lobe size as the response and another submodel with grazing damage as the response.

To identify the most informative microclimate predictors for each submodel, we used an information-theoretic model selection approach based on Akaike’s information criterion corrected for small sample sizes (AICc; Burnham and Anderson, 2002), implemented with the MuMIn package (Bartoń, 2025). Candidate environmental predictors included GDD above 5 °C, which is directly related to temperature, maximum VPD, which is related to air humidity, and canopy openness (%) as a measure of light availability (Table 1). These predictors were selected based on ecological relevance to lichen physiology and ecology (e.g. Greiser *et al.*, 2021) and low collinearity (*r* ≤ 0.7, *P* > 0.05; Dormann *et al.*, 2013). We fitted linear mixed models (LMMs) with Gaussian error for the final size submodels (final lobe area, biomass and STM) and generalized linear mixed models (GLMMs) with binomial error distribution and a logit link function for the grazing damage submodels. All full submodels included fixed effects of initial lobe size (area, biomass and STM, measured at the start of the experiment), population origin, the three environmental predictors (GDD, VPD and light) and their interactions with population origin. Grazing damage was included as a predictor in the final size submodels. By including initial lobe size along with the other predictors, we accounted for differences in size at the start of the experiment and thus the effects of other predictors on final size can be interpreted as effects on growth. Population origin was retained in all submodels to test for differences in responses among populations. Transplant site was included as a random intercept to account for the data structure. All continuous predictors were scaled by subtracting the mean and dividing by the standard deviation, except grazing damage, which ranged from 0 to 1 and had the same values when used as a response or a predictor. Submodels with ΔAICc < 2.0 were considered to have similar explanatory power and were used to calculate the averaged submodel and the relative importance of the predictors (Burnham and Anderson, 2002).

**Table 1.** Environmental and biotic predictors used in the lichen final size and grazing damage submodels of transplanted *Peltigera aphthosa* lobes across 56 forest sites in central Sweden during the growing season in autumn 2016 and spring–summer 2017.

Predictor	Abbreviation	Mean ± s.d.	Min.	Max.
Growing degree days (unitless)	GDD	1171 ± 119	983	1525
Maximum vapour pressure deficit (kPa)	VPD	0.73 ± 0.08	0.58	0.94
Canopy openness in summer (%)	Light	27.2 ± 6.1	13.5	49
Proportion of grazed tissue (%)	Grazing damage	30.2 ± 20	0.5	70

The averaged submodels resulting from the selection procedure were fitted using maximum likelihood estimation with the lme4 package (v.1.1.33) (Bates *et al.,* 2015). We checked that the variance inflation factor (VIF) for each submodel were <2 using the check_collinearity function in the package performance (v.0.8.0; Lüdecke *et al.*, 2021). The *P*-values for fixed effects were calculated using Satterthwaite’s degrees of freedom approximation with the lmerTest package (Kuznetsova *et al.*, 2017). We reported marginal and conditional *R*^2^ values to quantify the variance explained by fixed effects alone and by both fixed and random effects (Nakagawa and Schielzeth, 2013). The residuals of the model were checked for normality and homoscedasticity using the DHARMa package (Hartig *et al.*, 2024). The residuals of the final lobe size submodels showed heteroscedasticity, so we estimated 95 % bootstrap confidence intervals (10 000 iterations) using the bootMer function in lme4 (Bates *et al.,* 2015). Predictors were considered significant when the confidence intervals did not overlap zero. As bootstrap estimates closely matched the original model estimates, we kept the latter for simplicity.

Finally, the three piecewise SEMs were constructed including the hypothetical causal paths between the predictors of the submodels of final lobe size and grazing damage. We used Fisher’s *C* values to assess goodness of fit of the piecewise SEMs (Lefcheck, 2016) and conducted Tukey post hoc tests to evaluate the differences among the levels of the factor population origin.

## RESULTS

**Fig. 3.**
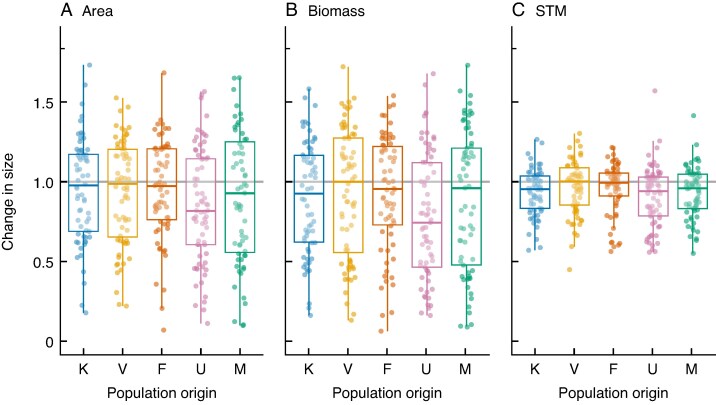
Changes in the size of the transplanted lobes of *Peltigera aphthosa* across 56 forest sites in relation to their population origin, ordered in decreasing latitude (K, Kiruna; V, Vindeln; F, Fagersta; U, Uppsala; M, Malexander; for their locations see Fig. 2B). For visualization, growth is represented as the proportional change in (A) lobe area (final area/initial area), (B) biomass (final biomass/initial biomass) and (C) specific thallus mass (final STM/initial STM). Values >1 indicate growth and values <1 indicate shrinkage.

The weather of the growing season in autumn 2016 and spring–summer 2017 was warm but not exceptional relative to the 1961–90 reference period (Greiser *et al.*, 2021; SMHI, 2024). More than half of the transplanted lobes (56 %) decreased in area and biomass during the experiment (Fig. 3A, B). Similarly, 58 % of the lobes exhibited a decrease in the STM, indicating thinning (Fig. 3C). Grazing damage was substantial (30 % on average), with only 16 % of the lobes showing minimal or no grazing marks (Table 1), and varied across transplant sites (Supplementary Data Fig. S2).

### Direct and indirect (grazing-mediated) effects of microclimate on lichen growth

**Fig. 4.**
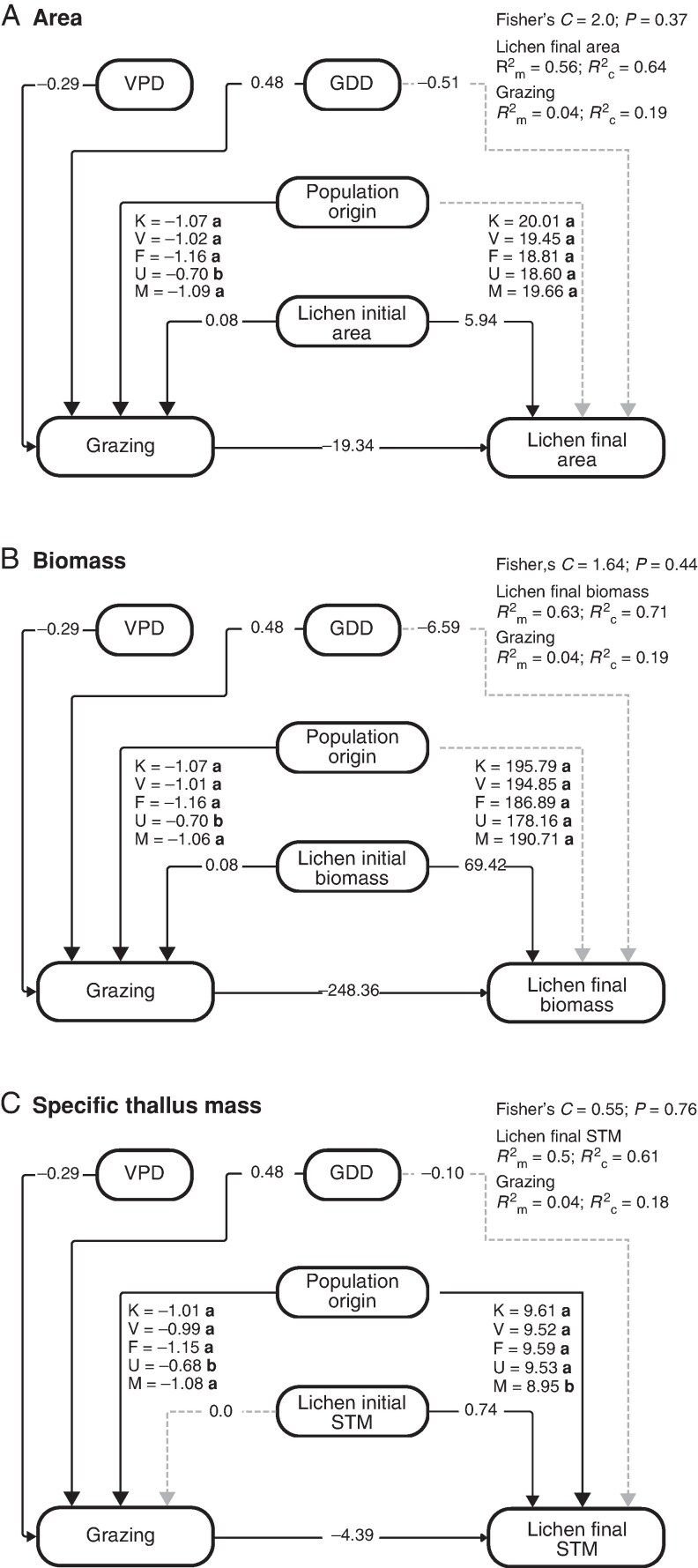
Results of piecewise SEMs examining the relationships among final lichen (lobe) size, initial lichen (lobe) size, microclimate predictors (GDD at 5 °C; maximum VPD), grazing damage and population origin (K, Kiruna; V, Vindeln; F, Fagersta; U, Uppsala; M, Malexander). The models include (A) final area (cm^2^), (B) final biomass (mg) and (C) final STM (mg cm^−2^). Transplant site was included as a random effect. Significant paths (*P* < 0.05) are shown as solid arrows with standardized coefficients, while non-significant paths are shown as dashed arrows. Marginal and conditional *R*^2^ values (*R*^2^_m_ and *R*^2^_c_), representing the proportion of variance explained by the fixed effects alone and by both fixed and random effects, respectively, are reported in italics for all response variables. The value of Fishers’ *C* statistic and its *P*-value are shown for each model. Different bold letters indicate significant differences (*P* < 0.05 in Tukey *post hoc* tests) in the effects among source populations.

From the initial set of environmental predictors, only GDD was selected as an informative predictor of final lobe area, biomass and STM, with growth increasing in colder sites (Supplementary Data Table S2a–c). In the grazing damage model, both GDD and VPD were selected, with grazing damage being greater at warmer and more humid sites (Supplementary Data Table S2d). Piecewise SEMs did not detect direct effects of GDD on lichen growth (Fig. 4). Instead, GDD influenced growth through grazing damage, which decreased lichen growth at warmer (higher GDD) and more humid (lower maximum VPD) sites (Fig. 4, Supplementary Data Table S2d). Grazing damage was also higher in larger lobes (Fig. 4A, B). Light availability (canopy openness) was not selected as an important predictor of either growth or grazing damage (Supplementary Data Table S2).

### Population-specific responses to microclimate and grazing damage

We found no evidence for differences in responses to microclimate among populations (interactions between population origin and environmental factors were not selected in the final model; Supplementary Data Table S2). However, the origin of the population influenced the final lobe area, biomass and STM through differences in grazing damage (Fig. 4, Supplementary Data Table S2). The mid-southern population (Uppsala) experienced significantly higher grazing damage, and therefore lower growth, than the other four populations (Figs 3 and 4). The southernmost population (Malexander) had significantly lower STM, both before and after transplantation (Fig. 4C, Supplementary Data Table S2).

## DISCUSSION

Our results, based on a replicated transplant experiment across field common gardens, revealed that the indirect effects of microclimate, mediated by mollusc grazing, were stronger than direct climatic effects on the performance of the cold-adapted lichen *P. aphthosa* near its warm-range margin. Our findings advance the understanding of the complex relationships between climate and antagonistic interactions and their combined effects on species performance, and have important implications for predicting species responses to climate change and for guiding conservation priorities.

### Absence of direct effects of microclimate on lichen growth

Contrary to our initial hypothesis, we did not detect direct effects of temperature or humidity on the growth of *P. aphthosa*. This result was unexpected given previous studies reporting rapid growth rates in this species (Dahlman *et al.*, 2002; Palmqvist *et al.*, 2017) and evidence of direct microclimatic effects on both transplanted and natural lichen populations (e.g. Larsson *et al.*, 2012; Merinero *et al.*, 2015; Stanton *et al.*, 2023). However, our findings are consistent with other experimental studies that incorporated biotic interactions and similarly found no direct climatic effects on plant performance (e.g. Welk *et al.*, 2014; Tomiolo *et al.*, 2015). Taken together, these studies suggest that climate-driven biotic interactions can mask or override direct physiological responses to climate.

In our experiment, transplanted lobes experienced varying levels of grazing, and direct climatic effects were not detected even in those not severely grazed. One possible explanation is that, although *P. aphthosa* can grow rapidly, the range of climatic variation captured in our study may have been insufficient to elicit effects on growth. While the microclimatic gradient was broad for most predictors (e.g. GDD), it was narrower for others (e.g. VPD), potentially constraining detectable physiological responses in *P. aphthosa*. This lack of response contrasts with patterns observed in other cold-adapted bryophytes and lichens transplanted at the same sites, where warmer and more humid conditions enhanced their growth, indicating that the microclimatic gradient was sufficient to trigger physiological responses (Greiser *et al.*, 2021). It is important to consider that our sites were located near the species warm-range margin, where conditions are relatively mild compared with more extreme parts of the distribution range, such as the cold-range margin, where direct physiological constraints on growth are likely to be more pronounced. If the experiment had encompassed a broader climatic gradient, it is possible that the direct effects of climate on growth would have been relatively more important. Nevertheless, the effects of microclimate on grazing damage indicate that indirect effects of climate via mollusc grazing are important for lichen performance near its warm-range margin.

Alternatively, lichen growth may plateau under certain environmental conditions, such that further increases in temperature or humidity do not result in additional area expansion or biomass accumulation. *Peltigera aphthosa* may also possess adaptive traits, such as efficient water uptake, protective pigments and flexible photobiont associations (including green algae and nitrogen-fixing cyanobacteria), which buffer it against moderate climatic variation. These traits could promote stable growth across diverse conditions, thereby reducing sensitivity to direct climate shifts. This pattern could reflect limited functional trait flexibility, as observed across other environmental gradients (Asplund and Wardle, 2015; but see Kershaw and Macfarlane (1980) for responses to light extremes). Supporting this interpretation, we observed differences in STM among populations before transplantation (with the southernmost population showing thinner lobes), but these differences remained unchanged after the experiment (Fig. 4C), suggesting that these traits are relatively fixed.

Overall, our results suggest that even fast-growing lichens at their warm-range margin may be more vulnerable to climate-mediated shifts in grazing pressure than to direct climatic effects. To further disentangle the direct effects of climate on lichen growth from those mediated by grazers, future multifactorial experiments should incorporate mollusc exclusion treatments. Besides, a multi-year experiment might have captured a broader range of weather variability, including climatic extremes such as prolonged drought, that could reveal stronger effects of microclimatic variation (e.g. Merinero *et al.*, 2020; Greiser *et al.*, 2021). However, extending the experiment was not feasible given the high grazing pressure observed in some sites, likely leading to the loss of lichen transplants and compromising the integrity of the dataset.

### Indirect effects of microclimate mediated by mollusc grazing on lichen growth

In agreement with our second hypothesis, elevated temperature and humidity reduced lichen growth by enhancing mollusc grazing. The observed increase in grazing damage under warmer and more humid conditions supports previous findings that antagonistic trophic interactions, such as herbivory, intensify with increased temperature and humidity (Rodríguez-Castañeda, 2013; Pepi and Karban, 2021; Lynn *et al.*, 2023). These microclimatic conditions likely promote greater local abundance, diversity and activity of lichen-feeding molluscs, thereby increasing the lichen consumption rate (Sternberg, 2000; Nicolai and Ansart, 2017). This is consistent with evidence indicating that warming exerts stronger direct effects on animal populations, such as increased growth rates and extended reproductive periods, than on plant populations (Hamann *et al.*, 2021; Kharouba and Yang, 2021).

The wide range of grazing damage observed in transplanted *P. aphthosa* across the 56 sites (ranging from 0.5 to 70 %) indicates that our study captured a wide gradient of grazing pressure. The average grazing damage across all transplant sites was substantial (30 %), suggesting that grazing may act as an ecological filter excluding the study species from some of the transplant sites. This is consistent with the study’s location near the species warm-range margin in Sweden, where mollusc diversity and abundance typically increase, intensifying antagonistic biotic interactions that potentially exert higher pressure on lichen populations (Gauslaa, 2008; Neubert *et al.*, 2019; Paquette and Hargreaves, 2021). However, most likely also other environmental factors and slow colonization rates contribute to the absence of the lichen species from forest patches (Sillett *et al.*, 2000; Werth *et al.*, 2006). Ultimately, our results contribute to growing evidence that near the warm-range margins of cold-adapted species, indirect climatic effects mediated through altered biotic interactions can more strongly influence species performance and distribution than direct climatic effects alone (Ockendon *et al.*, 2014; Hamann *et al.*, 2021; Christiansen *et al.*, 2024).

### Variation in responses to grazing among populations, but not to microclimate

Our results revealed no population-specific responses to microclimate, and thus no evidence of local adaptation to climate. However, populations differed in their susceptibility to grazing. We expected northern populations to be more negatively affected by grazing than southern populations, which are presumably better adapted to higher grazing pressure (Karban and Myers, 1989; Fornoni, 2011; Züst *et al.*, 2012). However, while we observed differences in grazing damage among populations, this prediction was not supported. The mid-southern population (Uppsala) experienced significantly higher grazing damage than others, suggesting that local adaptation to grazing pressure may not follow a simple latitudinal pattern. This population, which was the only one occurring on a rocky outcrop, may be more palatable due to differences in its chemical composition, potentially having fewer grazing-deterrent secondary metabolites and/or higher nutrient availability (i.e. lower C/N ratio) (Asplund and Wardle, 2013; Gadea *et al.*, 2019). This finding suggests that local environmental factors may be more influential than broad latitudinal gradients in driving population-level adaptation to grazing (Moles *et al.*, 2011; Moreira *et al.*, 2015). Additionally, larger lobes experienced more grazing damage, likely due to increased visibility or differences in chemical composition or nutrient content. This aligns with previous studies showing size-dependent herbivory in lichens (Benesperi and Tretiach, 2004). Understanding the local drivers of susceptibility to grazing requires further investigation of the specific environmental conditions of the source populations, including assessments of mollusc abundance and lichen chemical traits.

### Implications for species distribution under climate change

Our findings have important implications for predicting range shifts of cold-adapted species near their warm-range margin under climate change. Climate-driven antagonistic interactions, such as grazing by lichen-feeding molluscs, may constrain the ability of species such as *P. aphthosa* to persist at their current warm-range margins. In Sweden, climate projections indicate warmer temperatures, especially milder winters in the north, together with increased winter and spring rainfall and higher drought risk in the south (Andréasson *et al.*, 2004; Eklund *et al.*, 2015). These changes, particularly milder and wetter winters, are expected to increase mollusc abundance and activity, potentially expanding their range northward and to higher elevations (Sternberg, 2000; Willis *et al.*, 2006; Baur and Baur, 2013; Nicolai and Ansart, 2017). Evidence from southern Norway suggests that prolonged grazing seasons due to milder winters may already be limiting the warm-range margins of rare epiphytic lichens (Gauslaa, 2008). Consequently, the potential distribution of *P. aphthosa* can contract at its southern warm-range margin, although counterintuitive range shifts could occur through climate-induced changes in biotic interactions such as altered predator–prey relationships or habitat shifts in molluscs (Osmolovsky *et al.*, 2025).

The ecological responses of terrestrial molluscs to climate change in northern Europe remain poorly understood. Current evidence suggests species-specific responses, often mediated by vegetation composition (Nicolai and Ansart, 2017; Neubert *et al.*, 2019). Moreover, the observed variation in susceptibility to grazing among *P. aphthosa* populations indicates that population-level traits may modulate responses to biotic pressures throughout the species’ range. Our results highlight the need to incorporate climate-driven biotic interactions and intraspecific variation among populations into predictive models. Assuming uniform herbivore pressure or homogeneous population responses may lead to overestimates of future range expansions (Wisz *et al.*, 2013; Peterson *et al.*, 2019).

Identifying the relative contributions of direct and indirect climate effects on species performance is essential to guide conservation priorities. Understanding where and when climate-driven antagonistic interactions intensify can help prioritize populations most at risk, for example by identifying microrefugia less favourable to herbivores. Integrating the effects of microclimate on trophic interactions and species population differentiation into conservation planning will improve forecasts of species responses, particularly for cold-adapted species facing rapid climate change.

## Supplementary Material

mcag064_Supplementary_Data

## Data Availability

The data are available from Zenodo Repository (https://doi.org/10.5281/zenodo.19220263).

## SUPPLEMENTARY DATA

Supplementary data are available at *Annals of Botany* online and consist of the following. Table S1: environmental characteristics of the source populations of *Peltigera aphthosa* used in the transplantation experiment. Table S2: results of the averaged mixed linear models exploring the effects of initial lichen size, microclimate predictors and origin of population on the lichen final size. Figure S1: grazing damage categories. Figure S2: mean percentage of mollusc grazing damage on *Peltigera aphthosa* transplanted across the 56 sites in central Sweden.
