# Supplementary material for: Grazing mediates microclimate effects on lichen performance near its warm-range margin
Source: Ann Bot. 2026 Mar 28;137(7):2173–83. doi: 10.1093/aob/mcag064 (PMC13319332; doi:10.1093/aob/mcag064)
Supplement: mcag064_Supplementary_Data [file mcag064_supplementary_data.docx]

**Supplementary Information**

**Grazing mediates microclimate effects on lichen performance near its warm-range margin**

# **Table S1.** Environmental characteristics of the source five populations of the cold-adapted ground-dwelling lichen *Peltigera aphthosa* used in the transplantation experiment ordered in descending latitude (decimal coordinates in WGS84). Altitude, Forest type, substrate and canopy openness were measured in situ. Climate data were extracted from the global data base Chelsa (Karger et al. 2017). GDD: Growing degree days > 5 º C; Mean and MaxVPD: mean and maximum vapour pressure deficit.

| **Population origin** | **Latitude** | **Longitude** | **Altitude (m a.s.l.)** | **Forest type** | **Substrate** | **Canopy openness (%)** | **Mean annual Temperature (ºC)** | **Annual rainfall (mm)** | **GDD** | **Mean VPD** | **Max VPD** |
| --- | --- | --- | --- | --- | --- | --- | --- | --- | --- | --- | --- |
| Kiruna | 67.83294642 | 20.41409131 | 425 | *Picea abies*, *Betula* spp. | Mossy forest floor | 85 | -0.5 | 520 | 634 | 0.27 | 0.60 |
| Vindeln | 64.2303044 | 19.78191079 | 192 | *Pinus sylvestris* | Mossy forest floor | 40 | 2.6 | 558 | 1029 | 0.33 | 0.73 |
| Fagersta | 59.90319075 | 15.92854143 | 162 | *Pinus sylvestris* | Mossy forest floor and rock. | 55 | 5.3 | 699 | 1293 | 0.39 | 0.78 |
| Uppsala | 59.78068806 | 17.57825956 | 36 | Open mixed forest (*P.sylvestris, P. abies, Betula*. spp) | Rocky outcrop | 90 | 6.2 | 566 | 1388 | 0.40 | 0.81 |
| Malexander | 58.03705501 | 15.28448029 | 165 | Open mixed forest (*P.sylvestris, P. abies, Betula*. spp) | Mossy forest floor | 70 | 6.3 | 573 | 1363 | 0.39 | 0.75 |

# **Table S2.** Results of the averaged mixed linear models exploring the effects of initial lobe size (Area _t_, Biomass _t_, STM _t_), microclimate predictors (GDD: growing degree days; VPD: maximum vapour deficit) and origin of population on the a) final lobe area (Area _t+1_); b) final lobe biomass (Biomass _t+1_); c) final specific thallus mass (STM _t+1_); and d) grazing damage of transplanted lobes of *Peltigera aphthosa* across 56 forest sites in central Sweden. The data comprise the t-test (*t*) statistic based on Satterthwaite’s method for the Gaussian models (final area, biomass and STM), the Wald-type Z statistic (*Z*) for logistic models (grazing damange), coefficients and standard error (SE) of the predictors included in the models, significance value (*P*), the variance and standard deviation (SD) of the random effect of site on the intercept, and marginal and conditional R^2^.

| **a)** |  | **Area _t+1_ (cm^2^)** | | | | |  | | |
| --- | --- | --- | --- | --- | --- | --- | --- | --- | --- |
|  |  | | |  |  | | | | |
| **Fixed effects** | **Coefficient (SE)** | | | ***t*** | | ***p*** | | | |
| (Intercept) | 19.4 (0.98) | | | 19.83 | | **<0.001** | | | |
| Area _t_ | 5.66 (0.36) | | | 15.83 | | **<0.001** | | | |
| GDD | -1.54 (0.73) | | | -2.09 | | **0.037** | | | |
| population (Kiruna) | 0.89 (1.04) | | | 0.85 | | 0.395 | | | |
| population (Malexander) | 0.37 (1.03) | | | 0.36 | | 0.720 | | | |
| population (Vindeln) | 0.19 (1.02) | | | 0.19 | | 0.852 | | | |
| population (Uppsala) | -1.73 (1.01) | | | -1.70 | | 0.089 | | | |
|  |  | | |  | |  | | | |
| **Random effects** |  | | |  | |  | | | |
| Intercept-Variance (SD) | 23.35 (4.83) | | |  | |  | | | |
| Observations | 334 | | |  | |  | | | |
| Marginal R^2^ / Conditional R^2^ | 0.38 / 0.63 | | |  | |  | | | |
|  |  | | |  |  | | | | |
| **b)** |  | **Biomass _t+1_ (mg)** | | | | | |  | |
|  |  | | |  |  | | | | |
| **Fixed effects** | **Coefficient (SE)** | | | ***t*** | | ***p*** | | | |
| (Intercept) | 195.03 (10.97) | | | 17.77 | | **<0.001** | | | |
| Biomass _t_ | 65.83 (3.91) | | | 16.83 | | **<0.001** | | | |
| GDD | -19.77 (8.57) | | | -2.31 | | **0.025** | | | |
| population (Kiruna) | 4.88 (11.07) | | | 0.44 | | 0.660 | | | |
| population (Malexander) | -3.53 (10.98) | | | -0.32 | | 0.748 | | | |
| population (Vindeln) | 1.51 (10.76) | | | 0.14 | | 0.888 | | | |
| population (Uppsala) | -28.09 (10.77) | | | -2.61 | | **0.010** | | | |
|  |  | | |  | |  | | | |
| **Random effects** |  | | |  | |  | | | |
| Intercept-Variance (SD) | 3349.3 (57.9) | | |  | |  | | | |
| Observations | 334 | | |  | |  | | | |
| Marginal R^2^ / Conditional R^2^ | 0.40 / 0.68 | | |  |  | | | | |
| **c)** |  | | **STM _t+1_ (mg/cm^2^)** | | | | | |  |
|  |  | |  | |  | | | | |
| **Fixed effects** | **Coefficient (SE)** | | | ***t*** | | ***p*** | | | |
| (Intercept) | 9.75 (0.20) | | 49.11 | | | **<0.001** | | | |
| STM _t_ | 0.73 (0.07) | | 9.68 | | | **<0.001** | | | |
| GDD | -0.33 (0.15) | | -2.21 | | | **0.031** | | | |
| population (Kiruna) | -0.09 (0.21) | | -0.42 | | | 0.671 | | | |
| population (Malexander) | -0.77 (0.21) | | -3.62 | | | **<0.001** | | | |
| population (Vindeln) | -0.20 (0.20) | | -0.97 | | | 0.335 | | | |
| population (Uppsala) | -0.42 (0.20) | | -2.07 | | | **0.040** | | | |
|  |  | |  | | |  | | | |
| **Random effects** |  | |  | | |  | | | |
| Intercept-Variance (SD) | 1.03(1.0) | |  | | |  | | | |
| Observations | 334 | |  | | |  | | | |
| Marginal R^2^ / Conditional R^2^ | 0.25 / 0.57 | |  | |  | | | | |

| **d)** |  | **Grazing damage** | | | | | | |  | | | | |  |  |  |
| --- | --- | --- | --- | --- | --- | --- | --- | --- | --- | --- | --- | --- | --- | --- | --- | --- |
|  |  | |  | | | |  | | | |  | | | |  |  |
| **Fixed effects** | **Coefficient (SE)** | | | ***Z*** | ***p*** | | | | |  |  |  |  |  |  |  |
| (Intercept) | -1.17 (0.12) | | | -9.39 | **<0.001** | | | | |  |  |  |  |  |  |  |
| Area _t_ | 0.08 (0.03) | | | 2.59 | **0.010** | | | | |  |  |  |  |  |  |  |
| GDD | 0.49 (0.14) | | | 3.59 | **<0.001** | | | | |  |  |  |  |  |  |  |
| VPD | -0.29 (0.14) | | | -2.18 | **0.029** | | | | |  |  |  |  |  |  |  |
| population (Kiruna) | 0.07 (0.09) | | | 0.77 | 0.660 | | | | |  |  |  |  |  |  |  |
| population (Malexander) | 0.07 (0.09) | | | 0.70 | 0.748 | | | | |  |  |  |  |  |  |  |
| population (Vindeln) | 0.12 (0.09) | | | 1.38 | 0.888 | | | | |  |  |  |  |  |  |  |
| population (Uppsala) | 0.46 (0.09) | | | 5.10 | **<0.001** | | | | |  |  |  |  |  |  |  |
|  |  | |  | | | | |  | | | | |  | |  |  |
| **Random effects** |  | |  | | |  | | | | | |  | | | |  |
| Intercept-Variance (SD) | 0.61 (0.78) | |  | | |  | | | | | |  | | | |  |
| N | 334 | |  | | |  | | | | | |  | | | |  |
| Marginal R^2^ / Conditional R^2^ | 0.04 / 0.19 | |  | | |  | | | | | |  | | | |  |

| **Category** | **Description** | **Mean percentage of grazed tissue** | **Example** |
| --- | --- | --- | --- |
| a | No grazing marks | 0.5 | 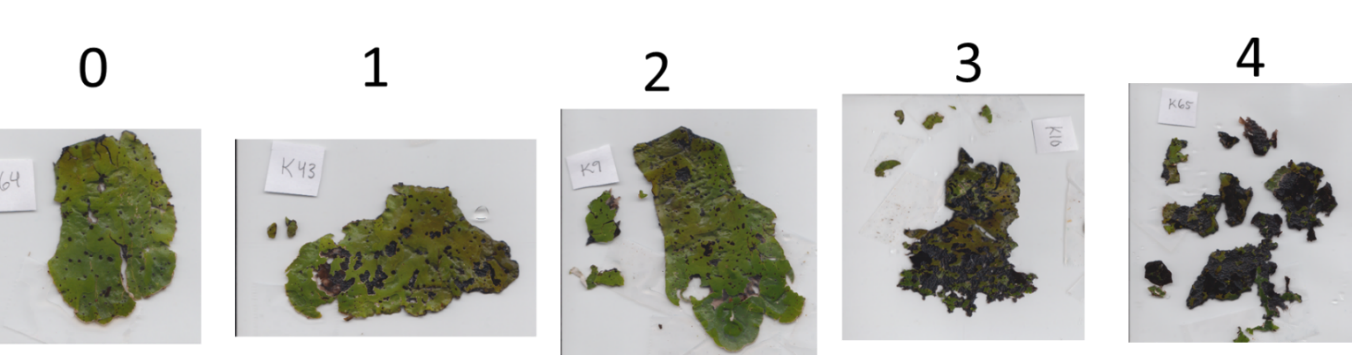 |
| b | Majority of grazing marks only on the upper surface | 6.5 | 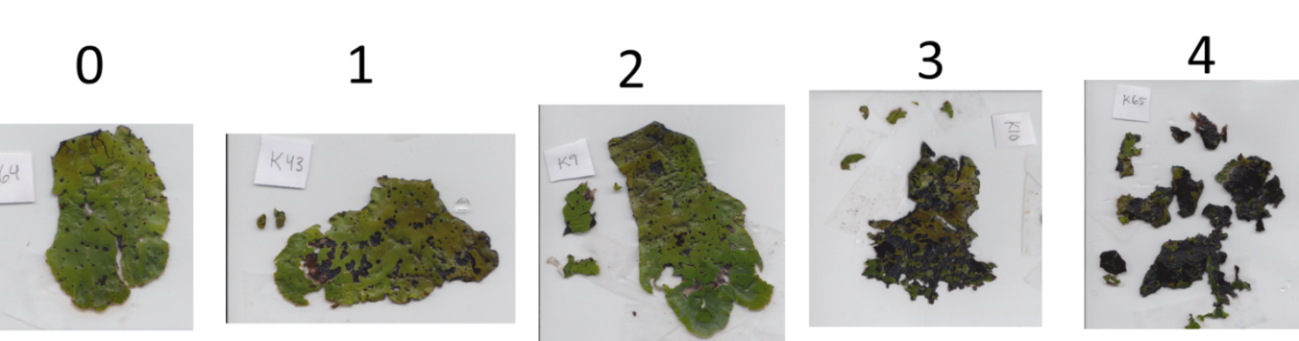 |
| c | Majority of grazing marks provoking area loss (i.e. holes and bites) | 15.5 | 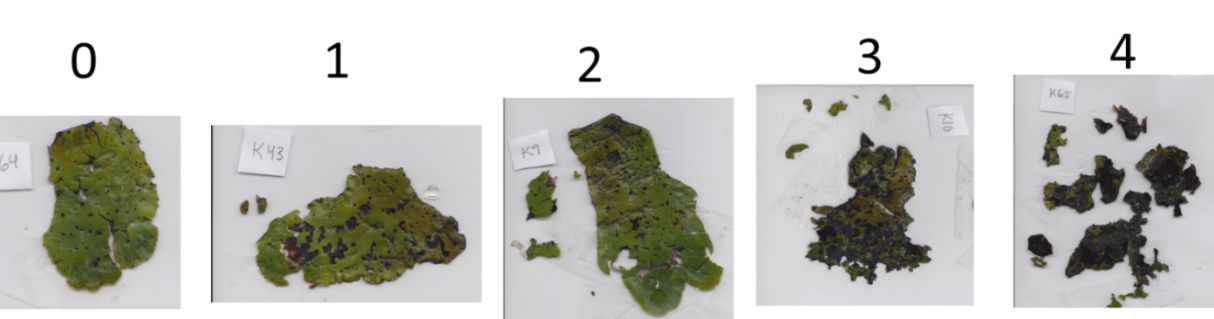 |
| d | Both bites marks and upper surface marks | 35 | 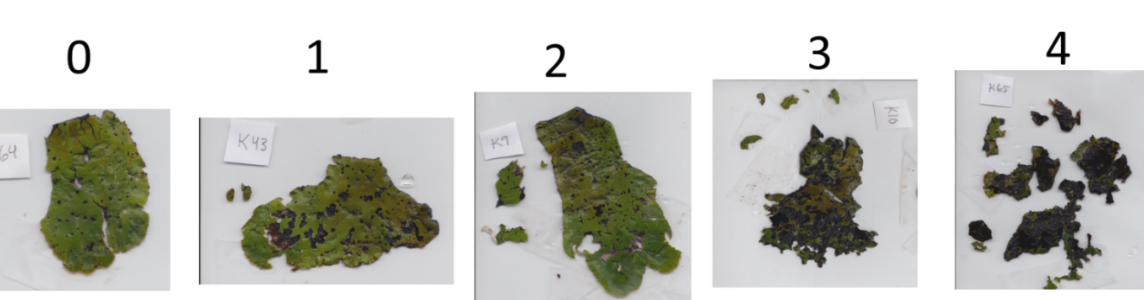 |
| e | Lichen components decomposition (black area >> green area). Not a functional lobe | 70 | 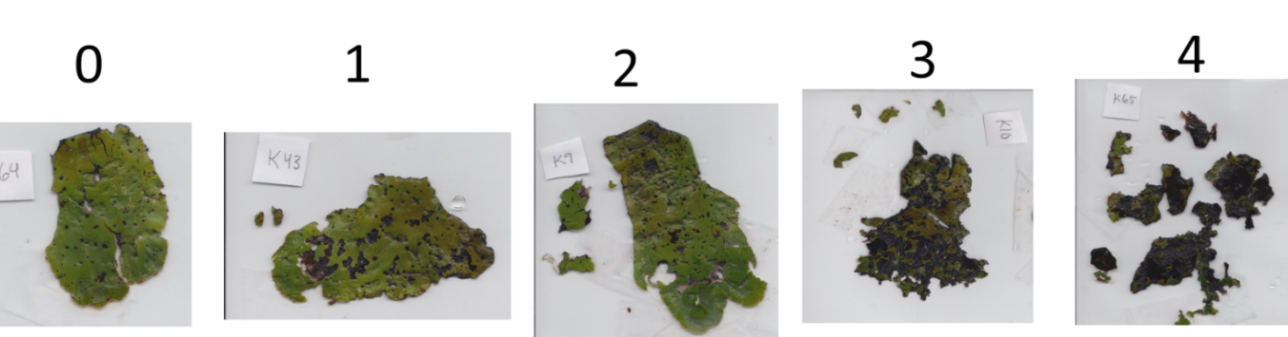 |

# **Figure S1.** Grazing damage categories.


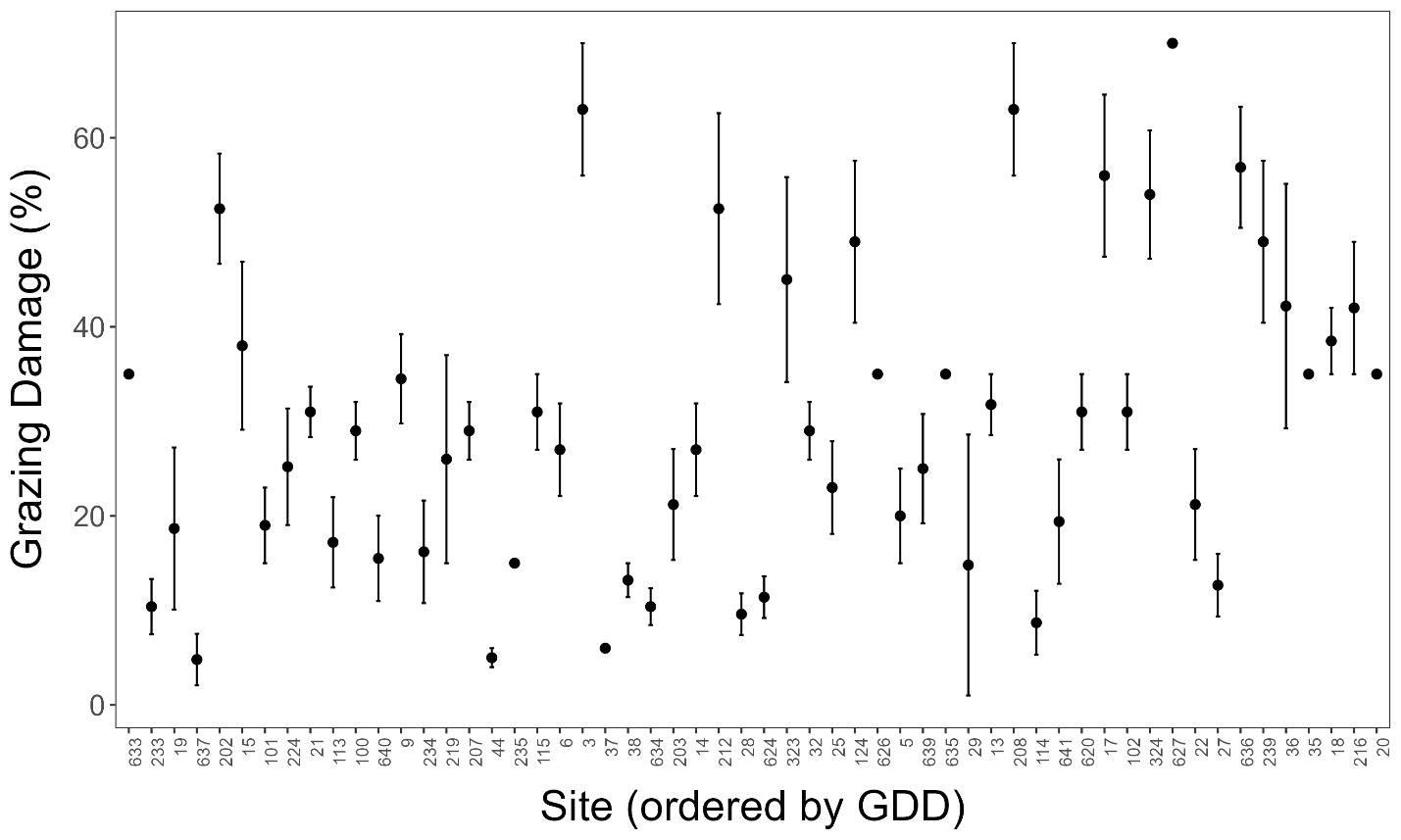


**Figure S2.** Mean (± SE) percentage of mollusc grazing damage on *Peltigera aphthosa* lobes (N = 334) transplanted across 56 sites in central Sweden. Each point represents the average damage per site (lobe number ranges from 1 to 10), illustrating spatial variation in grazing intensity. Sites are ordered by increasing temperature (GDD, growing degree days > 5 ºC).

**References:**

Karger, D.N., Conrad, O., Böhner, J., Kawohl, T., Kreft, H., Soria-Auza, R.W., Zimmermann, N.E., Linder, P., Kessler, M. (2017): Climatologies at high resolution for the Earth land surface areas.***Scientific Data*.**4 170122. <https://doi.org/10.1038/sdata.2017.122>
